# Predicting disease-related phenotypes using an integrated phenotype similarity measurement based on HPO

**DOI:** 10.1186/s12918-019-0697-8

**Published:** 2019-04-05

**Authors:** Hansheng Xue, Jiajie Peng, Xuequn Shang

**Affiliations:** 1 School of Computer Science, Northwestern Polytechnical University, Xi’an, China; 2 School of Computer Science and Technology, Harbin Institute of Technology, Shenzhen, China

**Keywords:** Human phenotype ontology, Semantic similarity, Phenotype similarity

## Abstract

**Background:**

Improving efficiency of disease diagnosis based on phenotype ontology is a critical yet challenging research area. Recently, Human Phenotype Ontology (HPO)-based semantic similarity has been affectively and widely used to identify causative genes and diseases. However, current phenotype similarity measurements just consider the annotations and hierarchy structure of HPO, neglecting the definition description of phenotype terms.

**Results:**

In this paper, we propose a novel phenotype similarity measurement, termed as *DisPheno*, which adequately incorporates the definition of phenotype terms in addition to HPO structure and annotations to measure the similarity between phenotype terms. *DisPheno* also integrates phenotype term associations into phenotype-set similarity measurement using gene and disease annotations of phenotype terms.

**Conclusions:**

Compared with five existing state-of-the-art methods, *DisPheno* shows great performance in HPO-based phenotype semantic similarity measurement and improves the efficiency of disease identification, especially on noisy patients dataset.

## Background

With the high-speed development of next generation sequencing (NGS) techniques, large-scale biological and medical data is generated exponentially, which greatly contributes to Mendelian disease and cancer diagnosis [1–3]. However, it is still difficult to make accurate clinic diagnosis solely based on sequencing technologies, because of the complex and incomprehensible relationships between genetic variants and clinical phenotypes [4].

Some observable features of patients, such as behaviors and biomedical properties, are defined as patient phenotypes, which are usually determined by both genetic and environmental factors [5]. Currently, patient phenotypes are widely used to improve efficiency of disease diagnosis by analysing the complex relationships between clinic phenotypes and phenotypes of known diseases.

**Fig. 1:**
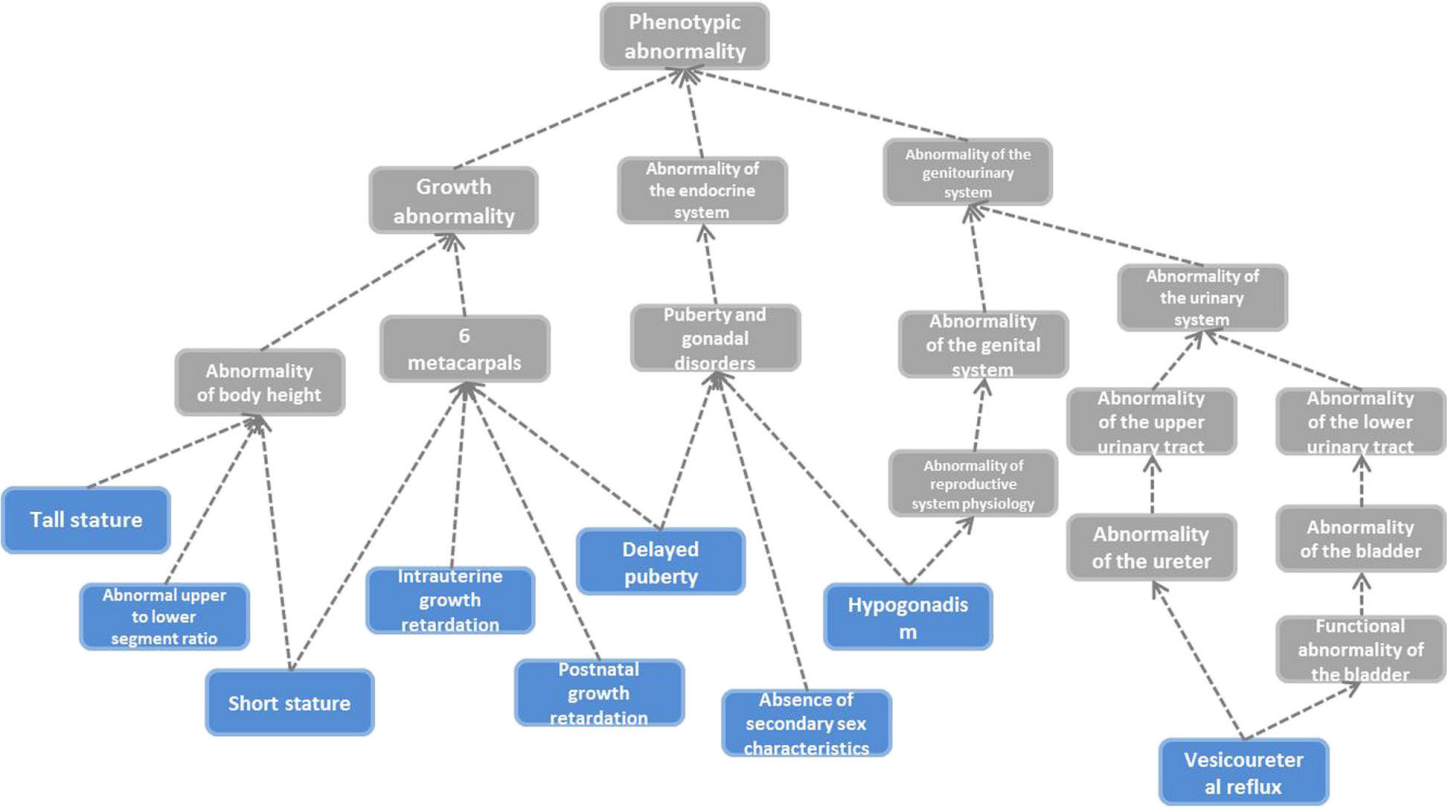
An illustrative example of Human Phenotype Ontology (HPO). An example of Phenotypic abnormality (HP:0000118) forming a directed acyclic graph (DAG), in which nodes represent phenotype terms and edges represent “subclass of ” relationships between phenotype terms

Human Phenotype Ontology (HPO) is a widely used ontology resource, which provides a standardized vocabulary of phenotypic abnormalities encountered in human disease [6]. HPO contains multiple types of information of phenotype, such as frequency modifier and definitions of phenotype terms. Besides, phenotype terms in HPO are organized as a directed acyclic graph (DAG) to describe the phenotypic characteristics and their relationships (An example is illustrated in Fig. 1). Based on HPO, researchers start to calculate phenotype similarity, which recently has been widely utilized to improve efficiency of disease diagnosis, and phenotype semantic similarity has become a rising research area [7, 8].

In phenotype semantic similarity area, previous researchers have proposed various HPO-based similarity measurements. Most of existing semantic similarity measurements are based on Information Content (IC), such as Phenomizer [9], OWLSim [10] and PhenomeNet [11]. In detail, Phenomizer measures any two phenotype terms similarity based information content of phenotype ontology, which is similar as Masino et al. [12]. PhenomeNet and OWLSim extend simGIC [13] to calculate phenotype similarity of two phenotype sets. However, IC-based similarity measurements ignore the associated relationships of phenotype terms. Besides IC-based measurements, most existing measurements are similar to GO-based similarity measurements and neglect the unique topological structure of HPO [14–22]. And the main difference between HPO and GO is the biological knowledge representing by their structure. In the low-level of GO structure, sibling terms are often similar to each other. In contrast, sibling terms in the low-level of HPO structure are hard to prove that they have associations at the gene level or share any disease symptoms. For instance, phenotype terms “Split hand (HP:0001171)” and “Areflexia of upper limbs (HP:0012046)” are two leaf terms in HPO, but between them, there is no known gene-level associations nor shared disease symptoms [23].

Thus, it is essential to propose a novel and unique HPO-based semantic similarity measurement which designs for considering topological information of HPO. We designed a new path-constrained IC-based phenotype term semantic similarity measurement, termed as *PhenoSim*, which considers the unique DAG structure of HPO [23]. In addition, some practical online or offline tools have been developed for biological researchers, including HPOSim [24] and PhenoSimWeb [25]. HPOSim provides an offline R package, which implements seven common ontology-based similarity measurements, including Jiang [26], Lin [27], Wang [28] and Schlicker [29]. PhenoSimWeb is an easy-to-use online application which implements five phenotype measurements and provides an intuitive visualization interface.

Although above methods are widely used to calculate phenotype semantic similarity, none of them make the best of phenotype ontology information, such as definition description of phenotype term and phenotype annotation information. *PhenoSim* proposed a phenotype similarity measurement based on topological structure of HPO, but it neglects text description and association information of phenotype term. Current HPO-based methods adopt gene or disease annotations to represent information content of phenotype term. However, this method cannot describe phenotype term fully and accurately, since many annotations associated with a phenotype are still unknown [30–32]. Therefore, it is essential and necessary to explore a novel phenotype similarity measurement that make the best of phenotype ontology information, such as hierarchical structure, term annotation and text description of phenotype.

In this paper, we propose a novel phenotype similarity measurement, named *DisPheno*, which integrates hierarchy structure and phenotype term definition of HPO. Compared with existing methods, the main contributions of our work can be summarized as:

- To the best of our knowledge, *DisPheno* is the first HPO-phenotype similarity measurement integrating term annotation, hierarchical structure and text description.
- *DisPheno* applies Point-wise Mutual Information to calculate phenotype annotations and integrates into phenotype-set similarity measurement.
- The evaluation results show that *DisPheno* outperforms some state-of-the-art approaches.

## Methods

**Fig. 2:**
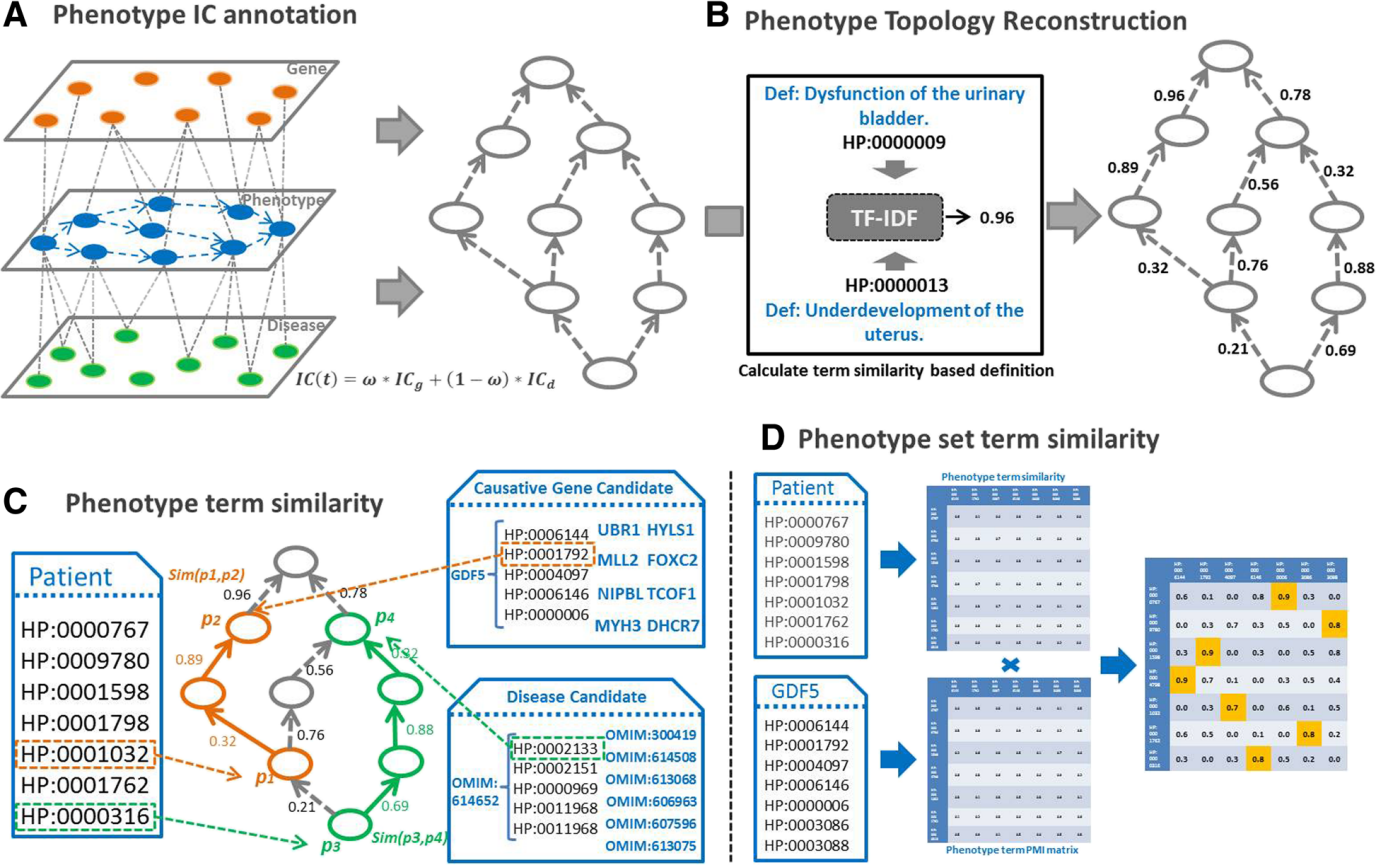
The workflow of DisPheno. It mainly contains four parts: **a** Annotating phenotype ontology information content using both gene annotation and disease annotation; **b** Reconstructing topological structure of phenotype term by calculating phenotype term definition similarity using TF-IDF; **c** Measuring phenotype semantic similarity based on HPO by integrating term definition-similarity; **d** Calculating phenotype term association and set similarity by measuring phenotype term associations using Point-wise Mutual Information(PMI)

In order to improve the performance of identifying disease-related phenotypes, we propose a novel phenotype similarity measurement, termed as *DisPheno*, which is a optimized method of a path-constrained information content-based similarity measurement. DisPheno mainly contains four steps. First, it annotates phenotype ontology information content using both genes and diseases. Second, it reconstructs topological structure of phenotype term using TF-IDF method [33]. Third, it computes semantic similarity between two phenotype term *t*_*i*_ and *t*_*j*_ considering information content(IC), distance between terms and DAG structure. Finally, it computes phenotype term associations using Point-wise Mutual Information (PMI) method [34] and calculates phenotype set similarity. The framework of *DisPheno* is shown in Fig. 2. and the detailed steps will be introduced as follows.

### Step 1. Annotating phenotype ontology information content

Most of current phenotype similarity measurement are based on information content(IC), and the types of annotating phenotype term mainly contains gene annotation and disease annotation. Existing phenotype similarity measurement are annotated using gene or disease, and our method integrates these two types of annotations. In annotating part, we use a weighted coefficient *w* to adjust the ratio of two types of annotations. The IC of phenotype term *t* can be described as follows:

$$IC(t)=w*{IC}_{gene}+(1-w)*{IC}_{disease}$$

$${IC}_{gene}(t)=ln\left(\frac{G}{G_{t}}\right) \qquad {IC}_{disease}(t)=ln\left(\frac{D}{D_{t}}\right) $$

where *IC*_*gene*_(*t*) represents the information content of phenotype term *t* annotated by genes, *G* and *G*_*t*_ represent the size of genes annotated to the root and term *t* respectively (*IC*_*disease*_(*t*) is similar to gene annotation). Finally, we can comprehensively integrate the relationships between phenotypes and genes / diseases into the information content of phenotype terms.

### Step 2. Reconstructing topological structure of phenotype term

Human Phenotype Ontology (HPO) provides a directed acyclic graph (DAG) to describe the phenotype term and associations. However, the edge of DAG has no weight and just indicate the hierarchical relationship. To further describe the relationship between phenotype terms, we try to turn original DAG into a weighted directed acyclic graph (WDAG). In our model, we calculate the cosine similarity between the definitions of phenotype terms using TF-IDF method and try to add weights for edges of original DAG.

To calculate the phenotype term similarity, we need to convert the term definition into vector by TF-IDF firstly. TF-IDF is short for term frequency-inverse document frequency, which is often used in data mining and information retrieval to measure the importance of a document in a collection or corpus [33].

Given a phenotype term definition *t*={*p*_1_,*p*_2_,...,*p*_*n*_}, *p*_*i*_ represents a specify word, and the term frequency of *p*_*i*_ is *tf*(*p*_*i*_,*t*)=*n*_*i*_/|*t*|, where *n*_*i*_ represents the times that word *p*_*i*_ occurs in phenotype term definition *t*, and |*t*| is the number of words in *t*. And the inverse document frequency of word *p*_*i*_ is $idf(p_{i},T)=\log {\frac {|T|}{|\{t\in T:p_{i}\in t\}|}}$, where |*T*| is the total number of phenotype term in the HPO corpus and |{*t*∈*T*:*p*_*i*_∈*t*}| is the number of phenotype term where the word *p*_*i*_ appears. Thus, the Term frequency-Inverse document frequency(TF-IDF) can be calculated as:

$$TF-IDF(p_{i},t,T)=TF(p_{i},t)*IDF(p_{i},T) $$

After translating the phenotype term definitions into TF-IDF vectors by calculating the word TF-IDF scores, we can calculate the term similarity between pair-wise phenotype term using cosine similarity based on the TF-IDF vectors. Then, we can obtain a phenotype term similarity matrix *S*∈*R*^*n*∗*n*^, where *n* is the number of total phenotype terms. Finally, we add the phenotype term similarity into the DAG and we can reconstruct the un-weighted directed acyclic graph into a weighted directed acyclic graph (WDAG). And the reconstructed WDAG will be used in the process of calculating phenotype term similarity.

### Step 3. Measuring phenotype semantic similarity

Most phenotype similarity measurements are based on information content, they just consider the information content of most informative common ancestor or phenotype terms. They neglect the effects of hierarchy structure and text description of phenotype terms.

In our previous research, *PhenoSim* has proposed a path-constrained information content-based phenotype similarity measurement. The core idea is to consider the structural accessibility of phenotype terms. In detail, if there is a directed path between any two phenotype terms *t*_*i*_ and *t*_*j*_ in the hierarchy structure of HPO, we consider that these two terms are highly similar to each other and “reachable”. Otherwise, these two phenotype terms are “unreachable” in the DAG structure of HPO.

Based on this measurement, we propose a novel method, termed as *DisPheno*, which considering the distance between term *t*_*i*_ and *t*_*j*_ and the pathway on the weighted directed acyclic graph. Thus, we define a novel phenotype-based similarity measurement as:

$$sim(t_{i},t_{j})=\left\{\begin{array}{ll} WIC(t_{MICA})*\left(1-\frac{dist(t_{i},t_{j})}{mostDepth}\right) & reachable \\ 0 & otherwise \end{array}\right. $$

where *WIC*(*t*_*MICA*_)= min(*IC*(*t*_*i*_),*IC*(*t*_*j*_))∗*W*(*t*_*i*_,*t*_*j*_), (*mostDepth-dist*(*t*_*i*_,*t*_*j*_))/*mostDepth* implies the influences of distance between *t*_*i*_ and *t*_*j*_, and *W*(*t*_*i*_,*t*_*j*_) is the weight product from *t*_*i*_ to *t*_*j*_ among weighted directed acyclic graph of HPO. Specifically, *mostDepth* describes the longest path in the hierarchy structure of HPO, or the maximum number of edges that leaf node reaches the root node.

### Step 4. Computing phenotype term association and set similarity

Before calculating the phenotype set similarity, we need to measure the association among all phenotype terms. Current phenotype set similarity measurements all adopt the average value of maximum phenotype term similarity between phenotype term and phenotype set as the phenotype set similarity. In our model, we introduce the phenotype association relationships and use Point-wise Mutual Information(PMI) to compute the phenotype term associations.

Assuming that if two term *t*_*i*_ and *t*_*j*_ belongs to same causative gene (or disease) in the gene-to-phenotype (or disease-to-phenotype) association file, we hold that term *t*_*i*_ and *t*_*j*_ are associated. The pair-wise association between phenotype terms can be calculated as:

$$PMI(t_{i},t_{j})=\log\left(\frac{p(t_{i},t_{j})}{p(t_{i})*p(t_{j})}\right)$$

where *p*(*t*_*i*_,*t*_*j*_) is the probability that term *t*_*i*_ and *t*_*j*_ appear on the same gene or disease annotation set simultaneously, *p*(*t*_*i*_) and *p*(*t*_*j*_) are total probability of term *t*_*i*_ and *t*_*j*_ in the phenotype annotation set.

Given a patient and a candidate gene(or disease), the corresponding phenotype sets are *T*_*p*_ and *T*_*c*_ respectively. The phenotype set similarity between specific patient and candidate genes (or diseases) are the average value of pair-wise phenotype terms similarities between *T*_*p*_ and *T*_*c*_:

$$\begin{array}{*{20}l} {Sim}_{set}(T_{p}\to T_{c})&=\frac{1}{N_{p}}\sum_{t_{i}\in T_{p}}\max_{t_{j}\in T_{c}}(sim(t_{i},t_{j})*PMI(t_{i},t_{j}))\\ {Sim}_{set}(T_{c}\to T_{p})&=\frac{1}{N_{c}}\sum_{t_{j}\in T_{c}}\max_{t_{i}\in T_{p}}(sim(t_{j},t_{i})*PMI(t_{j},t_{i})) \end{array} $$

where phenotype similarity *sim*(*t*_*i*_,*t*_*j*_) is measured in previous step and *N* described the number of phenotype terms in set *T*. Due to the similarity score relies on the order of the phenotype-set and the above two equation are asymmetric, we use the following equation to eliminate the asymmetry affects. The symmetrical phenotype similarity measurement are described as:

$${Sim}_{sym}(T_{p},T_{c})=\frac{1}{2}({Sim}_{set}(T_{p}\to T_{c})+{Sim}_{set}(T_{c}\to T_{p})) $$

Where *Sim*_*sym*_ is the average value of set similarities of two phenotype sets with different order. Phenotype term and set similarity measurement are the key of identifying true disease from candidate disease set. By modifying existing HPO-based similarity, we can further improve the efficiency of disease diagnosis.

## Results

### Data preparation

The experimental datasets were downloaded from Human Phenotype Ontology (HPO) official website (https://hpo.jax.org/), which contain 10,838 phenotype terms, 99,186 disease-to-phenotype annotations and 61,784 gene-to-phenotype annotations.

To evaluate the performance of our method, we used the patients that simulated in our previous work *PhenoSim*, which mainly contains “patients with known causative genes” and “patients with known diseases” two parts. Taking into account the clinical situation, we generated dataset with noise phenotype terms, named noisy dataset, and imprecision phenotype terms, named imprecision dataset. The optimal and noisy datasets used in this paper are same as our previous paper [35]. The details of simulating patients are described as follows.

**Optimal dataset** Each simulated patient was assigned one selected disease, and then we randomly added phenotype terms that selected disease associated with into this stimulated patient. In detail, if the randomly generated number was not greater than the known penetrance of the phenotype that disease associated with, this phenotype will be assigned to this simulated patient. The process was repeated for 100 times, then we obtained final optimal simulated patients.

**Noisy dataset** The noisy dataset is an extension of optimal, which considers the real clinic dataset. Before simulating noisy dataset, we firstly generated a noisy phenotype-set that much larger than the number of optimal phenotypes for every selected disease. The noise phenotype can be defined as the term which is not associated to this disease. After generating noisy phenotype-set, half number of noisy phenotype terms are selected and added into the phenotype set of simulated patients. Finally, we repeated this process for optimal patients and we generated the noisy simulated patients.

**Noisy & Imprecision dataset** Besides noisy phenotypes, clinical datasets usually contain imprecision phenotypes which attributes to the limitation of medical technology. The imprecision data is described as a kind of phenotype terms that one of their ancestors is associated with the disease *d* instead of the explicit phenotype term itself. In this noisy & imprecision dataset, we randomly selected half of the optimal terms and replaced them with one of their ancestors. Then we added noisy phenotype terms into the imprecision dataset, and the number of noise terms is half of the imprecision dataset. Finally, optimal, noisy and noisy & imprecision data all account for one-third of the whole dataset.

### Performance evaluation on optimal dataset

We utilized the same evaluation criterion with PhenoSim to validate the prediction performance of *DisPheno* [12]. The main idea is to rank the candidate diseases of each simulated patient. We calculated the phenotype similarity value between the patient and each candidate diseases using *DisPheno*, then ranked all the candidate diseases in descending order by their similarity values. Higher the true disease’s rank is, the better the algorithm’s performance. Finally, we compared *DisPheno* with other five existing state-of-the-art measures on all the simulated datasets.

**Fig. 3:**
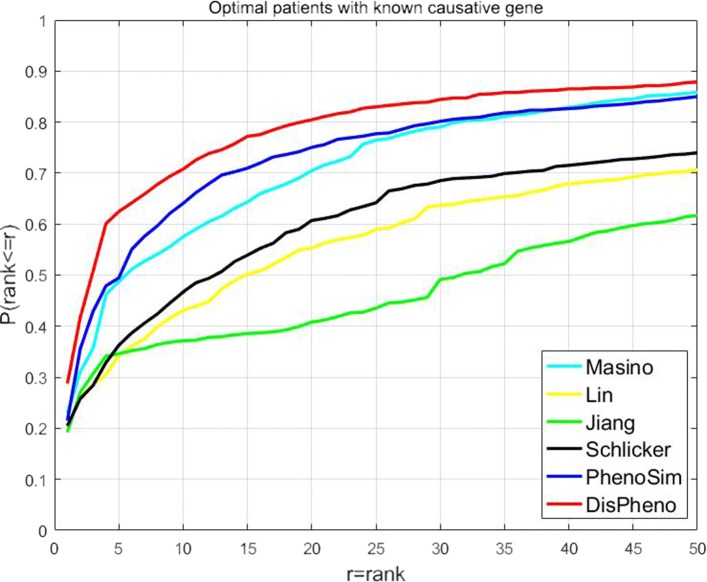
Cumulative rank distribution of optimal patient dataset with the known causative gene. The x-axis is the rank threshold and the y-axis is the cumulative probability of true disease rank

**“Optimal patients with known causative gene”** dataset contains 3300 simulated patients and each patient corresponds to one causative gene. We tested *DisPheno* and other five methods on this optimal dataset and compared the rank of true disease. Specifically, there is mapping relationship between causative genes and diseases. Because the HPO-based similarity measurements are usually used on disease diagnosis, we ranked the candidate diseases for each simulated patient instead of causative genes. In the cumulative rank distribution figure, we can find that *DisPheno* performed much better than the other methods (Fig. 3). 28.78% of true candidate diseases rank first using *DisPheno* which is the highest percentage among all methods. The percentage of rank among top-3 using *DisPheno* is 49.86%, while the ratio of other methods are 42.94% (PhenoSim), 35.69% (Masino), 28.55% (Lin), 30.69% (Jiang) and 28.42% (Schlicker) respectively. In addition, 60.43% candidate diseases rank among top-5 using *DisPheno* and it is 10.98% higher than *PhenoSim* (49.45%) which is the second best method.

**“Optimal patients with known disease”** dataset contains 24,000 simulated patients and each patient corresponds to one disease. We tested the performance of all six approaches on this optimal dataset (see Table 1). Although the percentage of top-1 using *DisPheno* (83.12%) is less than the ratio of Schlicker (96.36%), 99.10% of candidate diseases rank among top-3 which is the highest compared with other methods. Although top-1 percentage is not highest, *DisPheno* shows great performance on disease identification. In the clinical cancer diagnosis or disease prediction, it usually provides scientists with several top candidates instead of the single highest one.

**Table 1:** The percentage of cumulative rank distribution

Method	Top-1	Top-3	Top-5	Top-10
DisPheno	83.12%	99.10%	99.71%	99.87%
PhenoSim	79.50%	98.62%	99.45%	99.83%
Masino	82.48%	97.43%	98.63%	99.16%
Lin	95.68%	97.94%	98.63%	99.35%
Jiang	95.43%	98.17%	99.11%	99.69%
Schlicker	96.36%	98.31%	98.93%	99.53%

*DisPheno* was compared with other five methods on the optimal patient with the known disease

In the optimal datasets, *DisPheno* performs better than other five methods. And it also shows great performance and latent capacity on predicting disease and disease diagnosis. Considering that clinical phenotype set often contains lots of noise data, we further validate the performance of *DisPheno* on the simulated patient with noisy phenotype terms.

### Performance evaluation on noisy dataset

**Fig. 4:**
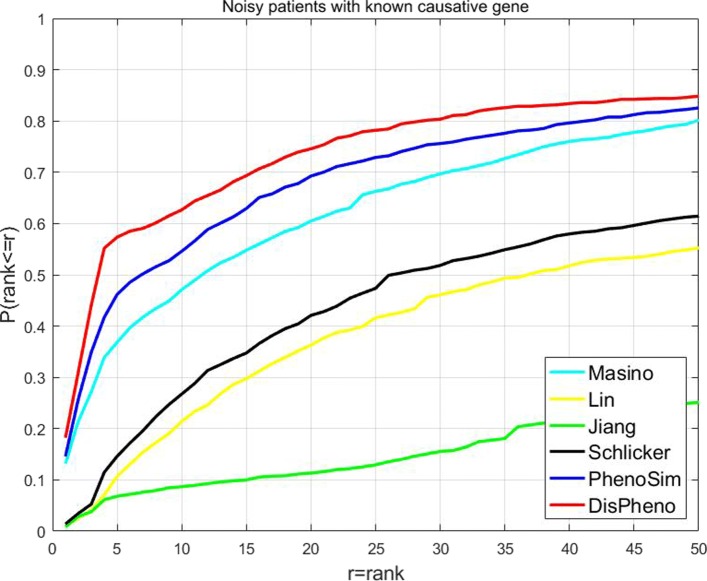
Cumulative rank distribution of noisy patient dataset with the known causative gene. The x-axis is the rank threshold and the y-axis is the cumulative probability of true disease rank

**“Noisy patients with known causative gene”** dataset contains noisy phenotypes which are not annotated phenotype terms of the causative gene. We applied *DisPheno* and other five measures on the noisy dataset. Our method performed the best in all the six measurements (Fig. 4). The ratio of true diseases rank among top-5 using *DisPheno* reaches the highest (57.38%), which is 11.20% higher than the second highest method PhenoSim (46.18%). The percentage of other methods perform on this dataset are 36.85% (Masino), 10.67% (Lin), 6.80% (Jiang) and 14.61% (Schlicker). *DisPheno* shows great performance on noisy patient with known causative gene, it indicates good application prospect on clinical diagnosis.

**Fig. 5:**
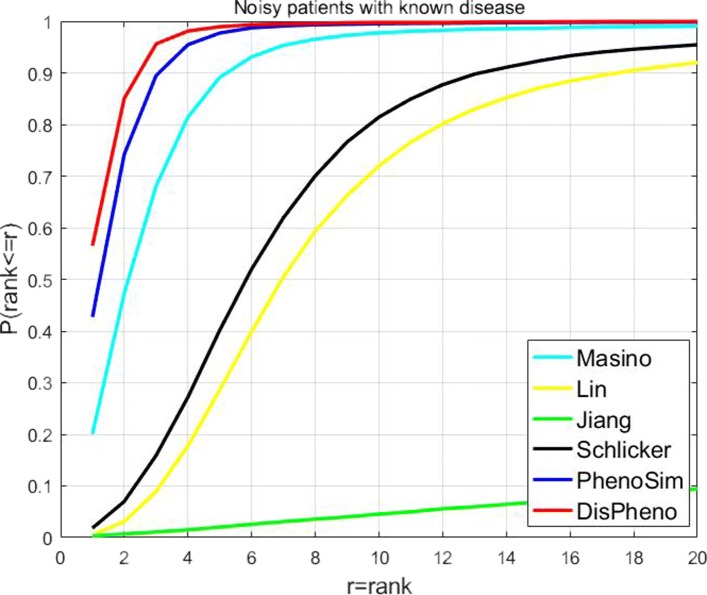
Cumulative rank distribution of noisy patient dataset with the known disease. The x-axis is the rank threshold and the y-axis is the cumulative probability of true disease rank

**“Noisy patients with known disease”** dataset contains noisy phenotypes which are not annotated phenotype terms of the disease. We applied *DisPheno* and other five approaches on the noisy dataset, and our method performed the best in all the six measurements (Fig. 5). On the noisy patients with known diseases, the performance of *DisPheno* is far superior than the other five algorithms. 56.48% of candidate diseases rank the highest using *DisPheno*. Instead, the ratio of other five methods are 42.74% (PhenoSim), 20.04% (Masino), 0.5% (Lin), 0.32% (Jiang) and 1.82% (Schlicker). The second highest is PhenoSim, which is 13.74% less than *DisPheno*. The great gap shows the performance of our method in disease identification, especially on noisy simulated patient dataset.

Overall, *DisPheno* performs better than other five similarity measurements on the stimulated datset with noise phenotype terms, and it shows great robustness. It implies huge potential on clinical disease diagnosis.

### Performance evaluation on noisy & imprecision dataset

Except noisy phenotype terms, clinical datasets often contains imprecision phenotypes. In this part, we performed *DisPheno* on the noisy and imprecision patient dataset with known disease to evaluate the performance respectively.

Compared with other five methods, *DisPheno* shows good and stable performance on simulated patients with noisy and imprecision phenotypes (see Table 2). The percentage of true disease rank among top-10 using *DisPheno* reaches 22.34%, which is much higher than others. It indicates that *DisPheno* would perform well on the clinical datasets and it shows great prospects on disease diagnosis.

**Table 2:** The percentage of cumulative rank distribution

Method	Top-10	Top-20	Top-30	Top-40	Top-50
DisPheno	22.34%	36.00%	44.55%	52.55%	58.07%
PhenoSim	3.86%	11.76%	20.69%	29.03%	36.49%
Masino	7.12%	23.76%	38.21%	48.89%	56.57%
Lin	2.14%	8.16%	15.16%	21.77%	27.98%
Jiang	1.66%	2.57%	3.45%	4.32%	5.25%
Schlicker	1.89%	6.88%	13.67%	20.34%	26.78%

*DisPheno* was compared with other five methods on the noisy & imprecision patient with the known disease

### Effects of parameters on DisPheno model

**Fig. 6:**
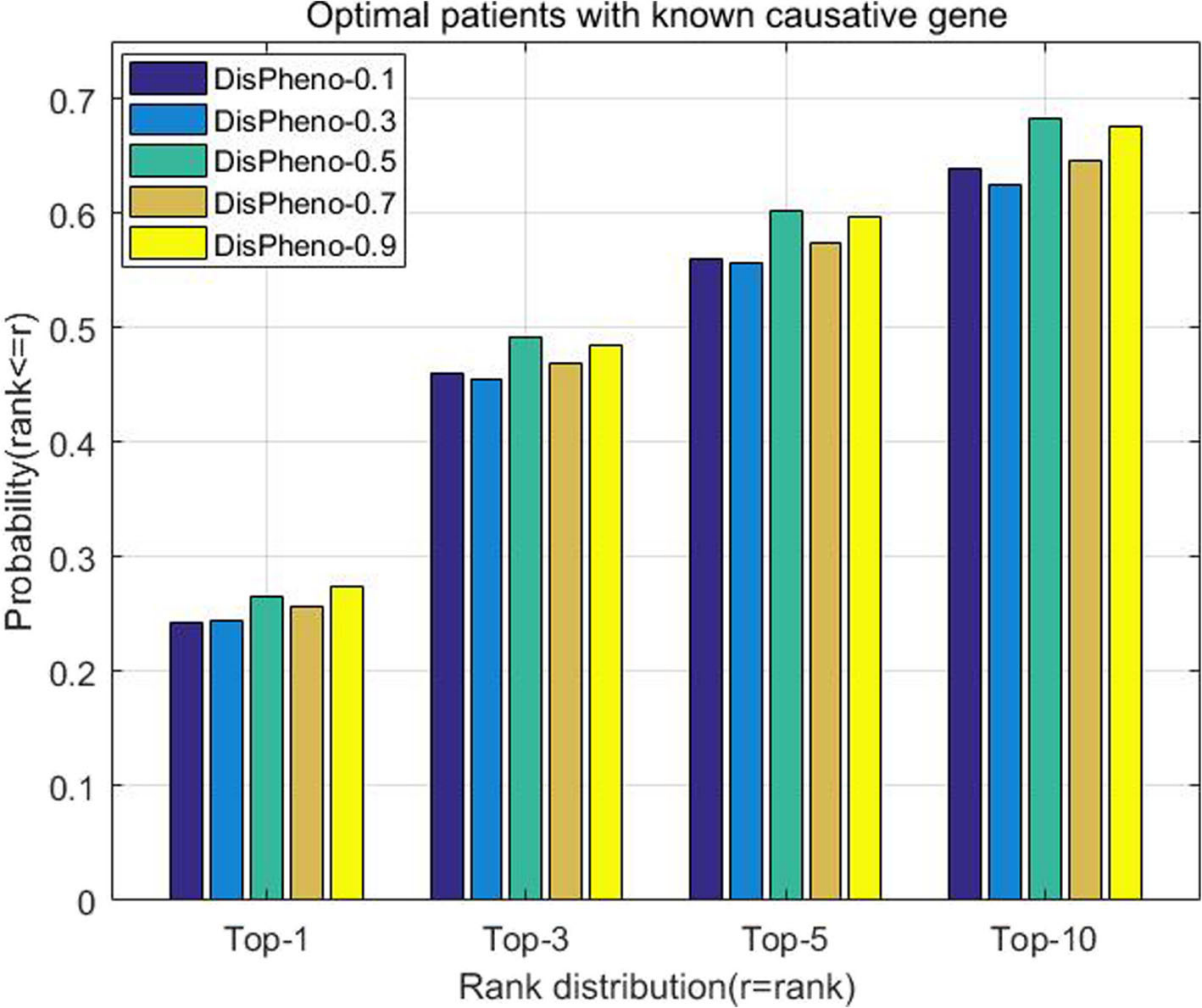
The histogram of cumulative rank distribution with different weighted coefficient between gene and disease annotations. The x-axis is the four different top ranks and the y-axis is the cumulative probability of true disease rank. When the weighted coefficient is 0.5 or 0.9, DisPheno achieves better performance on the optimal patients with known causative gene dataset

In this part, we test the various parameters on *DisPheno* model. In the first part of our model, we utilize both gene and disease annotations. We run *DisPheno* multiple times by varying the parameter *w* from 0.0 to 1.0 to test the performance of different weighted coefficients. Figure 6 shows that *DisPheno* achieves the best performance when the weighted coefficient is equal to 0.5 or 0.9.

**Fig. 7:**
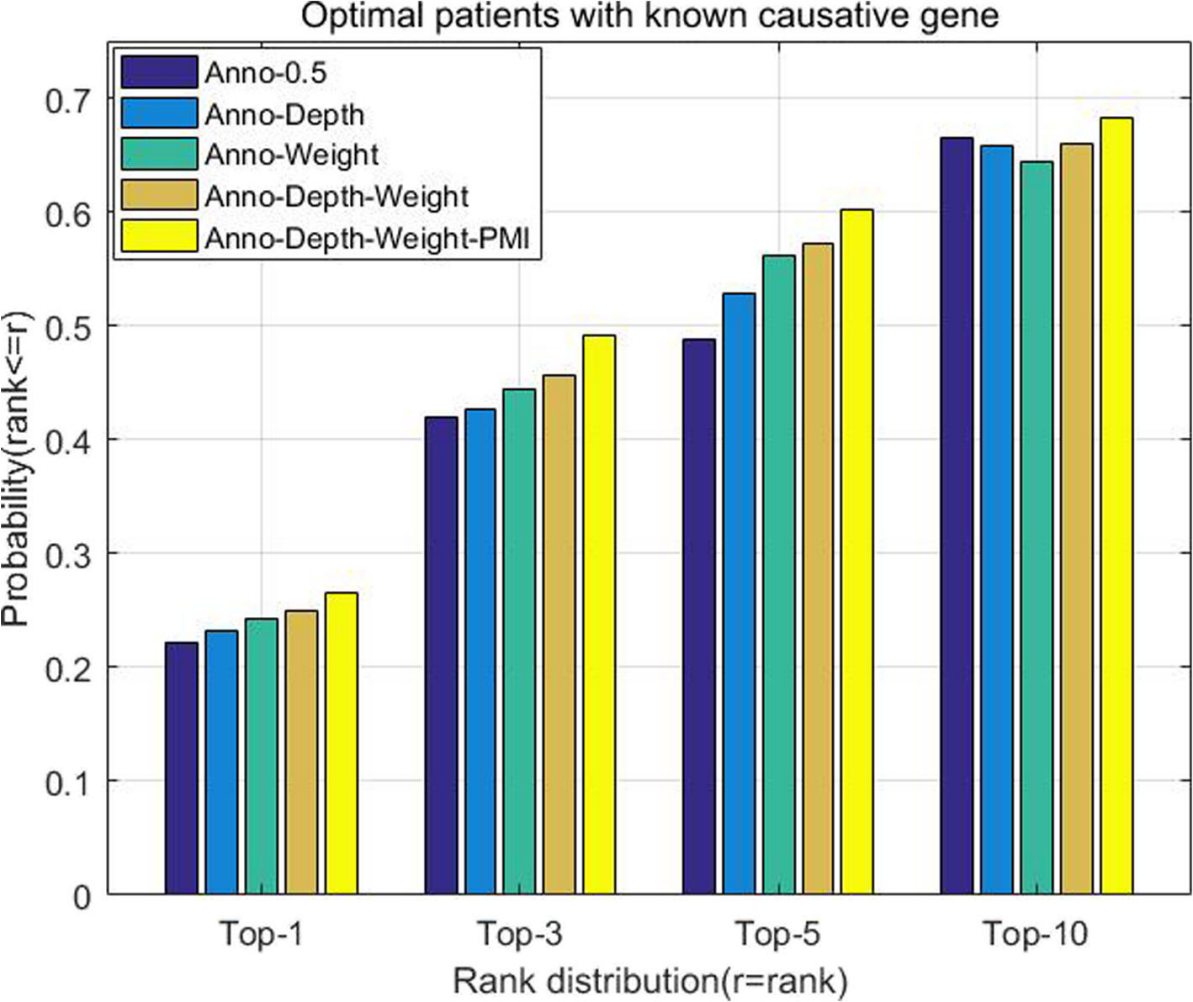
The histogram of cumulative rank distribution with different parts of *DisPheno*. The x-axis is the four different top ranks and the y-axis is the cumulative probability of true disease rank. The navy blue bar is *PhenoSim* method with part *Anno* and the weighted coefficient is 0.5. The sky blue contains part *Depth* based on previous step. The green one contains part *Weight*. The orange one combines part *Depth* and *Weight*. The yellow bar is the method *DisPheno* which performs better than others

Besides, we also run different parts of *DisPheno* to evaluate the contribution of different components in the model. Compared with previous algorithm *PhenoSim*, this novel model mainly adds four parts to improve the performance of identifying true disease. First part is utilizing both gene and disease to annotate phenotype terms, named as *Anno*. Second part of our model mainly consider the effect of the distance between two phenotype terms, thus we add (1−*dist*(*t*_*i*_,*t*_*j*_)/*mostDepth*) in the process of calculating phenotype term similarity, named as *Depth*. Besides, we utilize TF-IDF and Cosine Similarity to measure the similarity between any two phenotype terms based on their definitions. We then add term definition similarities into phenotype topological structure, and convert original directed acyclic graph into a weighted directed acyclic graph. This part is named as *Weight*. In the part of calculating phenotype term similarity, we calculate PMI matrix to measure the association of phenotype terms. This step is named as *PMI*. We run our model with different single part to evaluate the performance of *DisPheno*. Figure 7 shows that each part of *DisPheno* contributes to improve the performance of identifying true disease from disease candidate sets. From this experimental results, we can find that the phenotype annotation method, distance between two phenotype, definition of phenotype term and association of phenotype sets are all critical to phenotype similarity measure and it could significantly improve the performance of disease diagnosis.

### Performance evaluation on gene and disease similarity

To further test the performance of *DisPheno*, we also apply our method on similarity measurement of gene and disease. Each gene or disease can be annotated by a set of phenotype terms. Therefore, gene or disease similarity measurement can be translated into a task of measuring phenotype set similarity. We run our method *DisPheno* on a gene set and a disease set. Both of the two sets contain 20 genes or diseases. We use venn diagram to show the experimental results of five measurements (DisPheno, PhenoSim and other three methods randomly selected from Masino, Jiang, Lin and Schlicker). In detail, we firstly rank gene or disease pairwise similarities calculated by all five methods. Then, we calculate the intersection of top-20 gene pairs or disease pairs, and visualize the result by venn diagram.

**Fig. 8:**
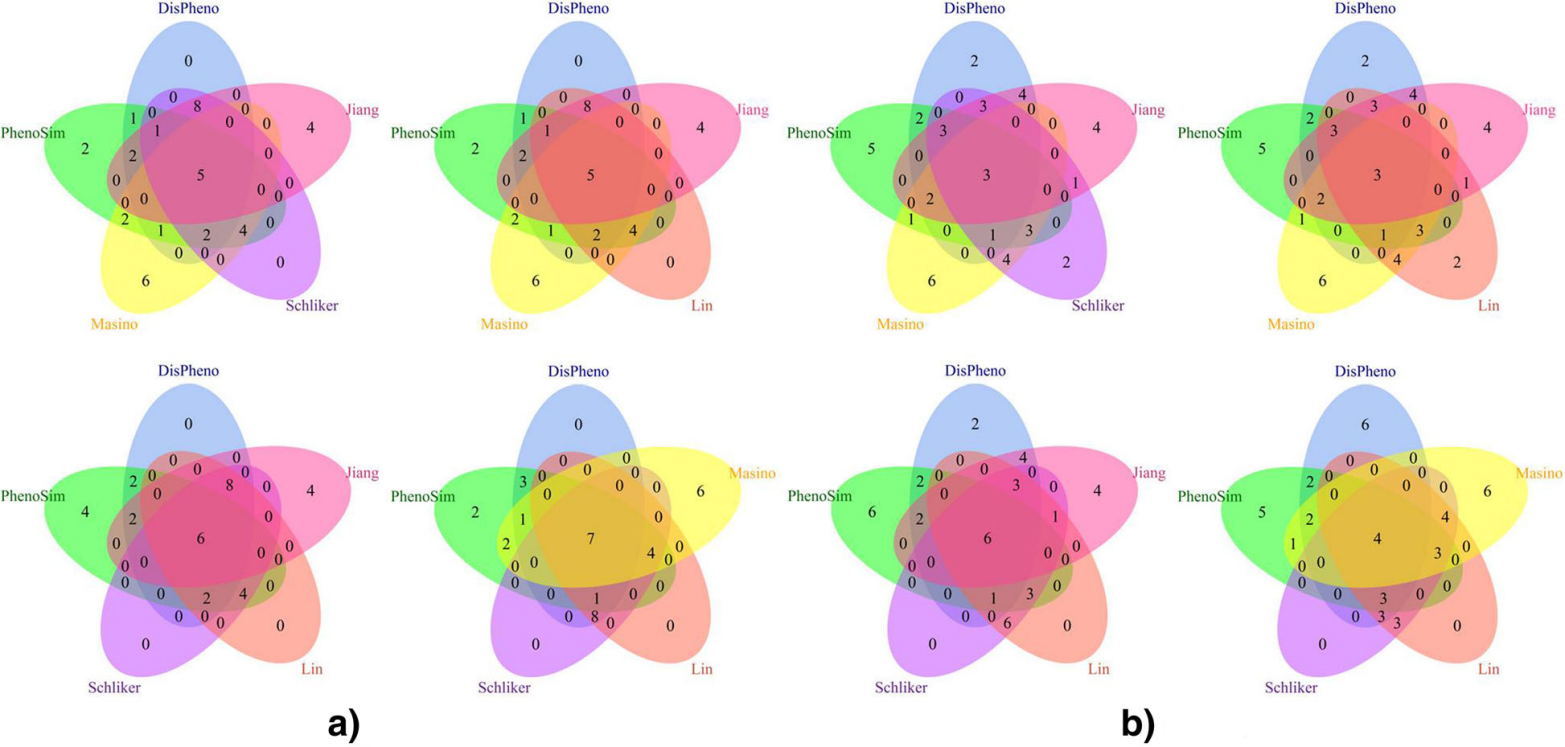
The venn diagram of top-20 gene and disease pairwise similarity. The blue and green are *DisPheno* and *PhenoSim*. The purple, tomato, yellow and red are *Schlicker*, *Lin*, *Masino* and *Jiang* respectively. From the intersection of venn figure, *DisPheno* performs better than other methods on task of gene and disease similarity measurement. For instance, the upper-left venn diagram shows that there are 5 pairwise genes are included in all methods’ results. All top-20 pairwise genes of DisPheno are contained by others. In contrast, there are 2 (PhenoSim), 4 (Jiang) and 6 (Masino) pairwise genes not belongs to any intersections. **a** Gene **b** Disease

The venn diagram (Fig. 8) shows that *DisPheno* is slightly better than other similarity measurements. We compare DisPheno and PhenoSim with other three methods which randomly selected from four phenotype similarity measurements. In the task of gene similarity calculation, the top-20 gene pairs of *DisPheno* are all part of others. In contract, *PhenoSim* contains 2 or 4 gene pairs which do not belong to any intersection. Similarity, *DisPheno* has fewer single disease-pairs than others in the task of measuring disease similarity.

**Fig. 9:**
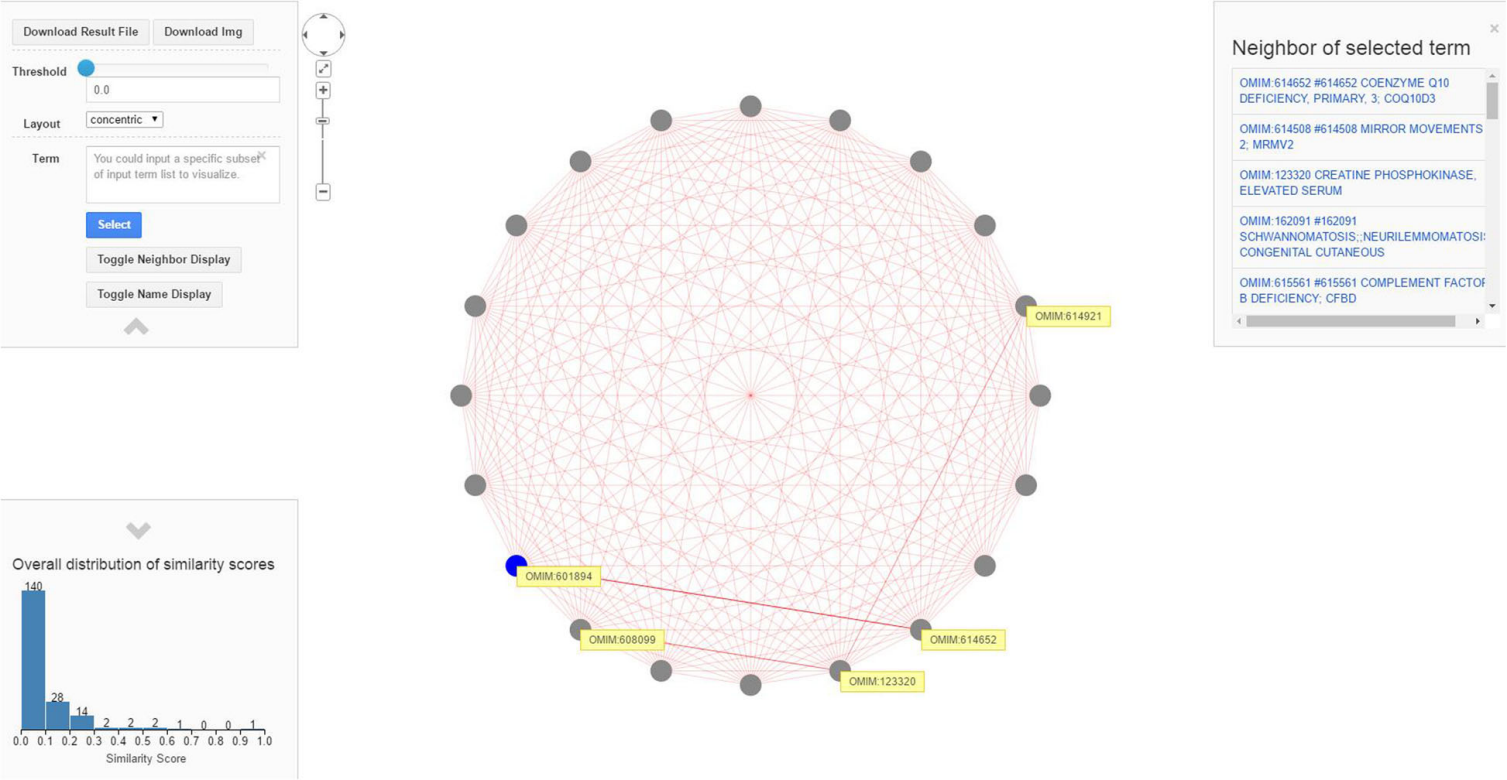
Disease similarity using visualization interface of *PhenoSimWeb*. We ran *DisPheno* on a disease dataset and calculated the pair-wise similarity of these diseases. Then, we visualized the results using the visualization interface of PhenoSimWeb. The main panel is the diseases association network, where nodes represent disease terms and edges represent similarities between diseases. The upper left is the mini control panel, where you can adjust threshold and choose different visual layout. The lower left part is the overall distribution of similarity scores. The upper right shows the neighborhood of selected disease term “OMIM:601894”

Besides, we used the visualization tool of *PhenoSimWeb* to visualize the disease and gene set similarity [25]. PhenoSimWeb is an online application which can be used to calculated phenotype, gene and disease similarity. It also can predict disease and causative gene based on the input phenotype set. PhenoSimWeb contains other useful tools, such as text description translator and visualization interface. And the visualization interface of disease set similarity calculated by *DisPheno* is shown in Fig. 9. The main panel is the terms association network, where nodes represent disease terms and edges represent similarities between diseases. The upper left is the mini control panel, where you can adjust threshold and visual layout. The lower left part is the overall distribution of similarity scores. The upper right shows the neighborhood of selected disease term “OMIM:601894”. This visualization webpage provides user a clear and convenient way to analysis the results of disease similarity.

*PhenoSimWeb* is an online phenotype similarity calculating and visualizing application, which currently contains five phenotype similarity measurements, including PhenoSim, Masino, Jiang, Lin and Schlicker. And in this paper, we propose a novel HPO-based phenotype similarity method. We will add our method *DisPheno* into the online tool *PhenoSimWeb* and enrich phenotype similarity measurement of this web application in the future.

## Conclusions

The high-speed development of biological techniques such as next generation sequencing has greatly improved efficiency of cancer prediction and disease diagnosis. However, intricate phenotype ontology and high genetic heterogeneity have stunted further improvement of disease identification. As an useful and powerful tool, HPO-based phenotype semantic similarity could fill this gap and accelerate the disease diagnosis effectively. In this paper, we proposed an unique and novel phenotype similarity measurement, called *DisPheno*, which integrates multiple types of information: hierarchical structure, phenotype term annotation and text description. Compared with existing five state-of-art methods on the optimal and noisy datasets, our method performs much better than the others. In summary, *DisPheno* accelerates the efficiency of disease identification significantly and it also shows greatly potentiality in practical clinical studies.

## Abbreviations

DAG: Directed acyclic graph
HPO: Human phenotype ontology
IC: Information Content
PMI: Point-wise mutual information
TF-IDF: Term frequency-inverse document frequency
